# Immune architecture of oocyte competence: from development to senescence

**DOI:** 10.3389/fimmu.2026.1968598

**Published:** 2026-10-20

**Authors:** Man-Xi Jiang, Yan Zhu, Huai L. Feng

**Affiliations:** 1 Center for Reproductive Medicine, The Affiliated Guangdong Second Provincial General Hospital of Jinan University, Guangzhou, China; 2 Department of Immunology, University of Pittsburgh, Pittsburgh, PA, United States; 3 New York Fertility Center, New York-Prebyterian Healthcare System Affiliate Weill Cornell Medical College, New York, NY, United States

**Keywords:** folliculogenesis, immune checkpoints, immunosenescence, macrophages, NLRP3 inflammasome, oocyte quality, ovarian aging, ovarian immune microenvironment

## Abstract

The mammalian oocyte does not develop in isolation. Throughout its protracted life history, from primordial germ cell specification to ovulation, the female gamete develops within a dynamic immune microenvironment that both supports and monitors its development. This review synthesizes current knowledge on the immunobiological mechanisms governing oocyte quality, with particular emphasis on how immune cells, cytokines, and inflammatory signaling pathways shape oocyte developmental competence across the reproductive lifespan. We examine the prenatal origins of ovarian immune colonization, the functional roles of tissue-resident macrophages, dendritic cells, and T lymphocytes in follicular dynamics, and the molecular pathways through which immune signals influence meiotic fidelity and mitochondrial function. The concept of the ovarian immune microenvironment as a critical determinant of oocyte quality is developed, integrating evidence from physiological ovarian aging and pathological conditions including primary premature ovarian insufficiency (POI). We further explore how immune checkpoint pathways, inflammasome activation, and senescence-associated secretory phenotype (SASP) converge to mediate age-related oocyte decline in preclinical models, with correlative human evidence supporting these observations. In preclinical models, these pathways have been associated with impaired oocyte quality through distinct mechanisms: spindle disruption and chromosome misalignment (TNF-α, IL-6), mitochondrial dysfunction and ATP depletion (NLRP3 activation, ROS), lipid peroxidation-induced membrane damage (ferroptosis), and impaired developmental competence manifesting as reduced fertilization and blastocyst formation rates (chronic inflammation); human evidence remains largely correlative. Emerging immunomodulatory strategies for fertility preservation are critically evaluated. This review conceptualizes oocyte quality as a property of immune-endocrine-stromal interactions and proposes microenvironment-aware models of female reproductive competence, while explicitly grading the strength of the underlying evidence.

## Introduction

### Oocyte quality: the central pillar of female fertility

Oocyte quality is the primary determinant of reproductive success in mammals. It encompasses the molecular and structural integrity required for fertilization, syngamy, and the initiation of embryonic development prior to zygotic genome activation (1). Unlike spermatogenesis, which proceeds continuously throughout male reproductive life, oogenesis is a finite and non-renewable process. The ovarian reserve, the pool of primordial follicles established perinatally, declines inexorably with age, and the quality of oocytes ovulated from this reserve deteriorates markedly after the fourth decade of life in humans (2–4). This dual decline in oocyte quantity and quality underpins the age-related reduction in female fertility and constitutes the major biological constraint on reproductive longevity. It is important to distinguish ovarian reserve, which denotes the size of the primordial follicle pool and thus the quantity of available oocytes, from oocyte competence, which refers to the functional potential of an individual oocyte to complete maturation, fertilize, and support preimplantation development. Although reserve depletion and competence decline are temporally correlated, they are not equivalent: a numerically adequate reserve can coexist with compromised oocyte competence, and vice versa. This review focuses on oocyte competence as the principal interpretive endpoint, while considering ovarian reserve as one of several functionally relevant determinants.

The assessment of oocyte competence has traditionally relied upon morphological criteria: cytoplasmic appearance, polar body extrusion, and meiotic spindle integrity. However, these subjective evaluations capture only the most overt manifestations of oocyte health and fail to reflect the complex molecular programs that determine developmental potential (5, 6). Multi-omics approaches have revealed that oocyte competence is encoded in transcriptomic, metabolomic, and proteomic signatures that are not apparent under light microscopy (7–9). Maternal-effect genes comprising the subcortical maternal complex, including *NLRP5*, *OOEP*, *TLE6*, and *PADI6*, exemplify this hidden molecular architecture, with biallelic variants in these components linked to recurrent preimplantation embryonic arrest in humans (10–12).

### The immune system: a reproductive regulator

Recent studies have substantially revised the relationship between immunity and reproduction. The immune system is now recognized as an active regulator of reproductive tissues, contributing to the cyclical remodeling, angiogenesis, and cell death that characterize ovarian function (13). Immune cells are not merely responders to infection but are integral components of the ovarian microenvironment, participating in every stage of follicular development from primordial follicle activation to corpus luteum regression (14).

This immune-ovarian dialogue operates through multiple modalities: direct cell-cell contact, paracrine signaling via cytokines and chemokines, and the transfer of extracellular vesicles (EVs) carrying microRNAs and proteins (15, 16). The ovarian immune microenvironment comprises a diverse repertoire of innate and adaptive immune cells, including macrophages, dendritic cells, natural killer cells, mast cells, and T and B lymphocytes. In ovarian tissue from patients with poor ovarian response, immune cells have been shown to account for approximately 52.6% of the total cell population, a proportion comparable to that of granulosa cells (17). By contrast, in IVF patients undergoing ovarian stimulation, leukocytes account for approximately 22% of follicular fluid-derived cells (18). The functional importance of these populations is evidenced by the reproductive consequences of their dysregulation: altered macrophage polarization, disrupted regulatory T cell (Treg)/T helper 17 cell (Th17) balance, and chronic low-grade inflammation have been reported in conditions of impaired fertility (19, 20).

This review synthesizes current knowledge on the immunological mechanisms that regulate oocyte development and quality in mammals. We adopt a life-course perspective, tracing the immune-oocyte relationship from prenatal ovarian colonization through postnatal folliculogenesis to the age-related deterioration of oocyte competence. We propose that oocyte quality is determined in part by the ovarian immune microenvironment, in addition to the oocyte’s intrinsic genetic and epigenetic status. We address four interconnected themes: (1) the prenatal establishment of the ovarian immune landscape and its implications for the primordial follicle pool; (2) the functional roles of immune cells in supporting follicular development and oocyte maturation; (3) the mechanisms by which immune dysregulation compromises oocyte quality in aging and disease; and (4) emerging therapeutic strategies that target the immune microenvironment to preserve fertility. Distinct from prior reviews (15, 21–23), the present work analyzes how the ovarian immune microenvironment may influence oocyte meiotic competence, mitochondrial function, and developmental potential, with oocyte quality as the primary interpretive endpoint and with explicit grading of the strength of evidence (1, 5, 6). We have organized the evidence according to functionally relevant endpoints—ovarian reserve, follicular growth, meiotic competence, and age-related decline—and distinguish direct oocyte endpoints from indirect ovarian endpoints and from evidence that remains preclinical or correlative. We focus on the mechanistic pathways of immunosenescence (inflammaging, NLRP3 inflammasome, and SASP) and ferroptosis that contribute to impaired oocyte quality, and we evaluate immunomodulatory strategies aimed at preserving oocyte developmental competence. We thereby propose a shift from oocyte-centric to microenvironment-aware models of reproductive competence.

Throughout this review, we integrate data from multiple experimental systems, each with distinct strengths and limitations. We have adopted a transparent evidence-grading framework: human-derived data from follicular fluid and ovarian tissue are presented as primary correlative evidence; data from peripheral blood or endometrium are cited as contextual clues only; rodent and non-primate models are discussed as preclinical experimental systems that provide mechanistic insight but require cautious extrapolation to human oocyte biology; interventional studies are distinguished from observational studies. All integrative models are working hypotheses, not established paradigms, and causal terminology is used only where supported by the corresponding level of evidence. For heuristic purposes, we retain the M1/M2 nomenclature and PI3K/AKT/mTOR pathway associations as operational shorthand for functional trends, not as definitive biological classifications, acknowledging that *in vivo* macrophage states exist along a continuous spectrum and that the same pathway can support either M1- or M2-like features depending on context (24, 25).

Furthermore, we organize the evidence around oocyte-specific endpoints rather than immune cell types or ovarian processes. For each immune mechanism, we specify: (i) the direct oocyte endpoint—a measurable property of the oocyte itself (meiotic spindle integrity, chromosome alignment, mitochondrial function, cytoplasmic maturation, fertilization competence, blastocyst formation); (ii) the indirect ovarian endpoint—a follicular or stromal parameter through which the immune signal secondarily affects the oocyte (follicle activation, granulosa cell function, vascular support, fibrosis, luteal function); (iii) the level of evidence, graded as described below; and (iv) human translational relevance, distinguishing human correlative data from preclinical mechanistic inference. **Table 1** provides a comprehensive evidence grading matrix classifying each mechanistic claim by species, tissue compartment, experimental design, directness to the oocyte, and translational maturity.

**Table 1:** Evidence grading matrix for mechanistic claims in ovarian immune-oocyte biology.

Claim/topic	Species	Tissue compartment	Experimental design	Directness to oocyte	Translational maturity	References
Macrophages promote primordial follicle activation via IL-1β, IL-6, TNF-α	Mouse	Ovarian (*in situ*)	Observational (diet-induced model)	Indirect (cytokines act on granulosa cells; oocyte effects inferred)	Preclinical; human validation needed	(41, 42)
CSF-1 deficiency impairs ovulation	Mouse	Systemic (global knockout)	Genetic knockout (interventional, preclinical)	Indirect (ovulation defect; oocyte quality not directly assessed)	Preclinical	(48)
TNF-α disrupts meiotic spindle organization	Mouse, Porcine	Isolated oocytes (ex vivo)	Direct cytokine exposure (interventional, preclinical)	Direct (oocyte-intrinsic effect)	Preclinical; human oocyte studies lacking	(52, 61)
IL-6 exposure causes spindle abnormalities	Mouse	Isolated oocytes (ex vivo)	Direct cytokine exposure (interventional, preclinical)	Direct (oocyte-intrinsic effect)	Preclinical; human oocyte studies lacking	(53)
Treg deficiency and Th1 shift in primary POI	Human	Peripheral blood + granulosa cells (*in vitro*)	Observational (case-control)	Indirect (systemic and *in vitro*; intra-ovarian Treg/Th17 mechanism not demonstrated)	Correlative; mechanistic studies pending	(67)
NLRP3 inflammasome activation in ovarian aging	Mouse	Ovarian tissue	Pharmacological inhibition (interventional, preclinical)	Direct (oocyte) + Indirect (tissue)	Preclinical; human correlative data exist	(75, 76, 80)
SASP and ovarian fibrosis	Mouse	Ovarian tissue	Pharmacological intervention (metformin; preclinical)	Indirect (fibrosis impairs follicle development; oocyte effects indirect)	Preclinical	(85, 86)
Ferroptosis in oocyte aging	Mouse	Isolated oocytes + ovarian tissue	Pharmacological inhibition (interventional, preclinical)	Direct (oocyte) + Indirect (tissue)	Preclinical; emerging	(91, 92)
Immune checkpoint expression in ovary	Human, Mouse	Ovarian tissue	Observational (expression profiling)	Indirect (expression documented; functional role unclear)	Correlative; functional studies pending	(66, 70)
Low-dose IL-2 for Treg expansion	Human	Peripheral blood (autoimmune disease; ovarian cancer)	Review of clinical trials (non-ovarian)	Indirect (mechanistic hypothesis; no ovarian functional data)	Non-ovarian clinical data only; ovarian application untested	(55)
MSC/EV therapies for ovarian rejuvenation	Mouse, Rat	Ovarian tissue	Cell/EV administration (interventional, preclinical)	Indirect (tissue-level effects; oocyte quality as secondary endpoint)	Preclinical; clinical evidence preliminary	(42, 56, 57, 102, 103)
PD-1/PD-L1 vs CTLA-4 blockade exert distinct ovarian effects	Mouse	Ovarian tissue	Pharmacological inhibition (interventional, preclinical)	Indirect (ovarian reserve/follicle endpoints; oocyte quality inferred)	Preclinical; human correlative data limited	(66, 70, 71)

This matrix classifies each mechanistic claim by species, tissue compartment, experimental design, directness to the oocyte, and translational maturity. Claims classified as “indirect” or “preclinical” should not be interpreted as established causal relationships in human oocyte biology.

## Immune regulation of ovarian reserve and primordial follicle pool

### Prenatal colonization and primordial follicle pool establishment

The establishment of the ovarian immune microenvironment begins during fetal development, concurrent with the formation of the ovarian reserve. In both humans and mice, the fetal ovary is colonized by immune cells derived from sequential waves of hematopoiesis (13). Primitive macrophages arising from the yolk sac seed the developing gonad before the establishment of definitive hematopoiesis, and these are subsequently supplemented or replaced by monocyte-derived fetal macrophages that infiltrate the ovary as the circulation develops (13, 26).

The timing of immune colonization coincides with critical events in gametogenesis. In mice, primordial germ cells migrate to the genital ridges between embryonic days 9.5 and 10.5, where they undergo mitotic expansion, cyst formation, and subsequent waves of apoptosis that eliminate the majority of germ cells (27). In humans, the peak germ cell number, approximately 7 million at mid-gestation, is followed by a dramatic reduction to 1–2 million oocytes at birth (28, 29). This attrition is not merely passive cell death but an active selection process in which surviving oocytes receive cytoplasmic and organellar contributions from dying germ cells, a phenomenon described as a “selection and nourishment” mechanism that ensures the quality of oocytes that enter the primordial follicle pool (30). The SDF-1/CXCR4 chemokine pathway, which guides primordial germ cell migration, exemplifies the shared molecular language between immune cell trafficking and germ cell navigation (31, 32). This shared chemokine axis suggests that the same guidance cues that direct immune cell homing may also shape germ cell positioning within the developing gonad, providing a molecular basis for immune-germline interaction during ovarian reserve establishment.

The role of immune cells in this prenatal selection process remains incompletely understood. However, macrophages localized in the fetal ovary exhibit profiles suggestive of angiogenic and tissue-remodeling functions (13, 26). They may contribute to the vasculogenesis that supports the developing gonad and to the breakdown of germ cell cysts that enables individual oocytes to associate with somatic pre-granulosa cells.

### Postnatal maturation and the quiescent follicle pool

Following birth, the ovarian immune microenvironment undergoes further maturation. Tissue-resident macrophages acquire specialized phenotypes that reflect their organ-specific functions, and the ovary becomes populated with adaptive immune cells, including T and B lymphocytes (26, 31). In the adult ovary, immune cells are distributed throughout the cortex and medulla, with particular enrichment in the theca cell layers of growing follicles and in the developing corpus luteum (32, 33). The relative abundance and composition of these immune populations shift as follicles advance from the primordial to the preantral and antral stages, with macrophage and T-cell densities increasing in the theca-stromal compartment alongside follicle growth (32, 34).

The postnatal maturation of the ovarian immune niche is shaped by both intrinsic developmental programs and environmental influences. The onset of puberty and the initiation of cyclical gonadotropin signaling induce profound changes in immune cell composition and activation status. Cyclic changes in ovarian steroid hormones such as estrogens and progesterone, directly modulate immune cell function, creating a dynamic immune environment that fluctuates across the estrous or menstrual cycle (35, 36). This immune-endocrine crosstalk is bidirectional: immune cells produce factors that influence steroidogenesis, while steroid hormones regulate immune cell recruitment, survival, and effector functions.

The primordial follicles that constitute the ovarian reserve reside in a relatively immune-sparse and poorly vascularized cortical niche within the ovarian cortex. This quiescent pool is characterized by limited vascularization, which restricts immune cell access and creates a microenvironment that protects resting oocytes from immune surveillance (13, 37, 38). As follicles progress to the preantral stage, they acquire a greater degree of immune privilege through the formation of the blood-follicle barrier, which further isolates the developing oocyte from systemic immune influences (38, 39). Notably, even within this immune-privileged preantral follicle compartment, granulosa cells are capable of bidirectional signaling with local immune cells, producing chemokines that recruit macrophages and T cells while responding to inflammatory cues (13, 40).

However, this immune privilege is not absolute. Macrophages localize in proximity to primordial follicles, and their secreted factors can influence the activation of follicular growth (13, 41). The transition from primordial to primary follicle, the first step in follicular recruitment, is associated with the local accumulation of macrophages and the transient production of proinflammatory cytokines including interleukin-1 beta (IL-1β), interleukin-6 (IL-6), and tumor necrosis factor-alpha (TNF-α) (41, 42). In preclinical models, these signals have been reported to activate phosphoinositide 3-kinase (PI3K)/Akt/mammalian target of rapamycin (mTOR) and Janus kinase (JAK)/signal transducer and activator of transcription 3 (STAT3) signaling pathways in oocytes and pre-granulosa cells, promoting the metabolic activation and cell cycle entry required for follicular growth (43, 44); direct evidence in human follicles remains limited. This inflammatory signal is tightly regulated and resolves rapidly; its persistence has been suggested to be detrimental to follicle integrity.

## Immune influences on follicular development and oocyte growth: direct and indirect oocyte endpoints

In this section, we evaluate how immune cells in the follicular microenvironment affect oocyte competence through direct oocyte endpoints (meiotic competence, mitochondrial function, cytoplasmic maturation) and indirect ovarian endpoints (follicle activation, granulosa cell function, vascular support); **Figure 1** illustrates the dual roles of macrophages across distinct ovarian compartments in physiological and pathological conditions, with the M1/M2 balance as a contributor to ovarian homeostasis and oocyte quality.

**Figure 1:**
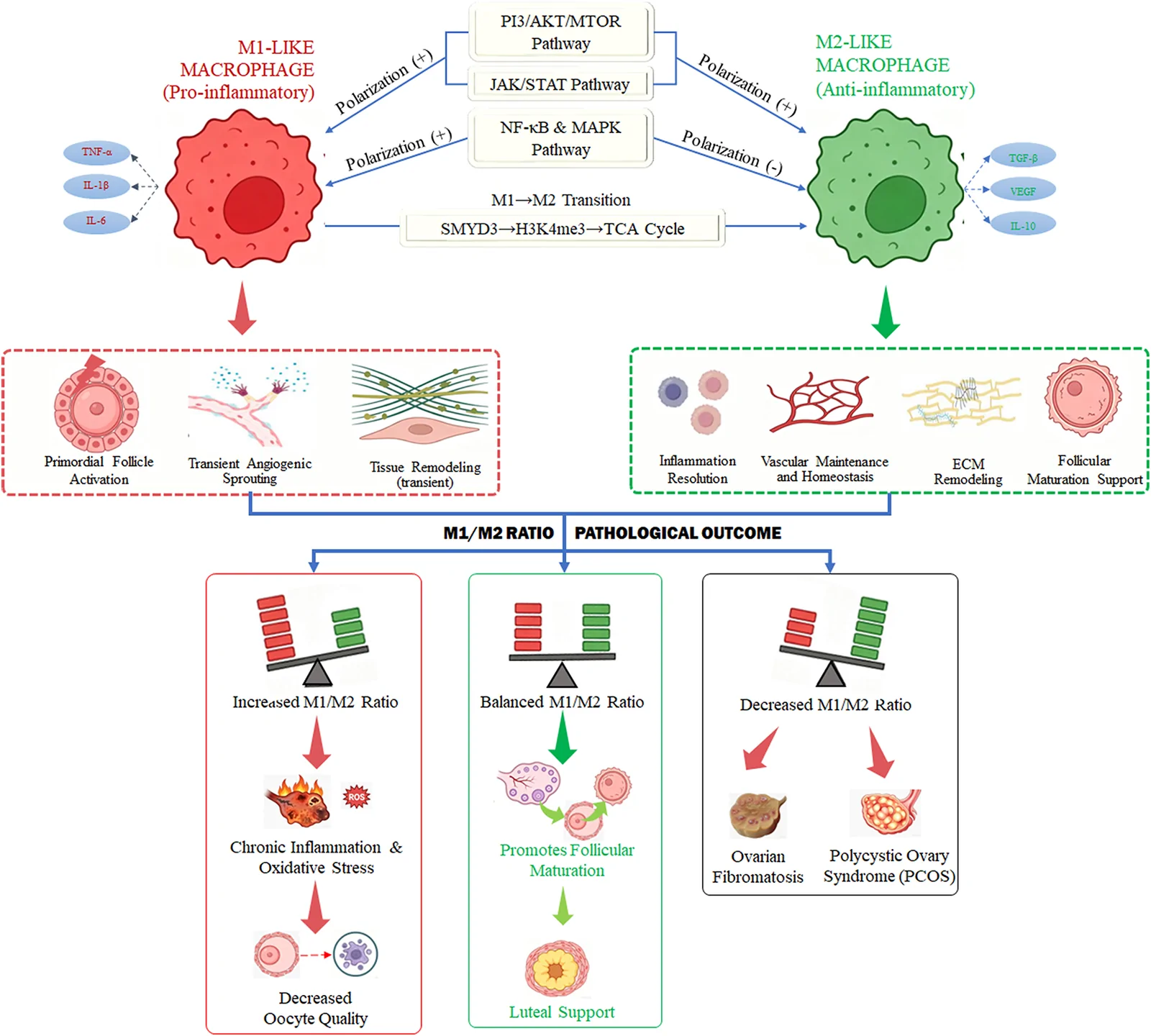
Macrophage polarization and its downstream effects in the ovarian microenvironment. This diagram illustrates the major signaling pathways and functional outcomes associated with M1-like (pro-inflammatory) and M2-like (anti-inflammatory/repair) macrophages in the ovary. M1-like polarization is primarily driven by the NF-κB and MAPK pathways, leading to the production of pro-inflammatory cytokines (TNF-α, IL-1β, IL-6), which have been associated with primordial follicle activation, transient angiogenic sprouting, and tissue remodeling—processes required for normal ovulatory function. The PI3K/AKT/mTOR pathway is depicted in association with M1-like phenotypes in this context, acknowledging its well-established role in metabolic reprogramming during inflammatory activation; however, its effect on polarization is context-dependent and can also promote M2-like features under different stimuli. Conversely, M2-like macrophages are frequently associated with the JAK/STAT pathway and an epigenetic–metabolic axis involving SMYD3-mediated H3K4me3 and TCA cycle activation, which together drive the production of anti-inflammatory mediators such as IL-10. These M2-associated activities contribute to inflammation resolution, vascular maintenance and homeostasis, extracellular matrix (ECM) remodeling, follicular maturation support, and luteal support, thereby facilitating post-ovulatory repair and cyclic ovarian function. While pro-inflammatory and reparative processes are both essential for physiological ovarian dynamics, their sustained or dysregulated activation may contribute to pathological conditions such as ovarian fibrosis. Reparative phenotypes are not uniformly antifibrotic, and persistent reparative signaling may, in some contexts, promote fibrotic remodeling. This schematic represents a simplified framework; the depicted pathways are not mutually exclusive, and significant crosstalk exists between them.

### Macrophages: key regulators of ovarian homeostasis

Macrophages are the most abundant immune cells in the ovary, constituting approximately 5-15% of follicular tissue cells in IVF patients (45), and serve as major regulators and key components of immune-ovarian interactions (40, 46). The distribution of immune cells, however, varies markedly across ovarian compartments and developmental stages. In the adult human ovary, leukocytes are distributed throughout the cortex and medulla, with particular enrichment in the theca cell layers of growing follicles and in the developing corpus luteum (32, 33). Within the primordial follicle pool—which resides in a relatively immune-sparse cortical niche—macrophages localize in proximity to quiescent follicles and can influence their activation (13, 41). As follicles progress through the preantral and antral stages, immune cell density increases in the surrounding theca-stromal compartment, with macrophages and T lymphocytes becoming more prominent (32, 34). This stage-dependent distribution underscores that immune-ovarian interactions are not uniform but are dynamically regulated according to follicular developmental status. The role of the somatic ovarian compartment—including its immune microenvironment—in reproductive function is illustrated by ovarian transplantation studies. Habermehl et al. demonstrated that transplantation of oocyte-depleted mouse ovaries into ovariectomized recipients restored endocrine function and supported fertility, effects that could only be attributed to the somatic stroma and its resident immune populations rather than to the germline (47). This finding provides direct experimental evidence that the ovarian somatic compartment, including its resident immune and stromal populations, can sustain reproductive competence in the absence of oocytes, although immune-specific depletion was not performed and the contribution of resident immune cells per se remains to be defined. Furthermore, the requirement for macrophages in female reproduction is supported by studies of colony-stimulating factor-1 (CSF-1) deficiency. Cohen et al. showed that CSF-1-null mice, which are severely deficient in tissue macrophages, exhibit disrupted estrous cyclicity and impaired ovulation, directly linking macrophage insufficiency to reproductive failure (48). These observations collectively indicate that macrophages and the broader immune stroma are important for normal ovarian function and follicle health in preclinical models, although their quantitative contribution to human oocyte competence remains to be defined. Importantly, recent single-cell RNA sequencing studies have revealed that ovarian macrophages are more heterogeneous than the M1/M2 binary model implies, with multiple myeloid subpopulations exhibiting mixed transcriptional signatures that do not conform neatly to either pole (24). As schematized in **Figure 1**, the PI3K/AKT/mTOR pathway is shown in association with M1-like polarization to reflect its well-characterized role in metabolic reprogramming during inflammatory activation; however, this assignment is context-dependent, and the same pathway can also support M2-like functions under alternative stimuli (25). The majority of ovarian follicles undergo atresia rather than ovulation, and macrophages clear the apoptotic debris from atretic follicles. This clearance function represents an additional facet of immune function in the ovary, distinct from the pro-inflammatory and reparative roles of macrophages described above (13, 40, 49, 50).

### Macrophages in primordial follicle activation: indirect ovarian endpoint with downstream consequences for oocyte competence

The direct oocyte endpoint here is not follicle activation itself, but the size and quality of the oocyte cohort that subsequently enters the growing pool; evidence for a direct effect on oocyte meiotic competence remains preclinical. The activation of primordial follicles determines the rate at which the ovarian reserve is depleted and is controlled by local and endocrine signals. Macrophages have been implicated in this process in preclinical mouse models; whether this translates to human ovarian physiology remains to be established. During the activation phase, cluster of differentiation 11c^+^ (CD11c^+^) macrophages transiently release IL-1β, IL-6, and TNF-α, which synergistically activate PI3K/AKT/mTOR (25) and JAK/STAT3 signaling in oocytes and pre-granulosa cells, reducing forkhead box O3 (FOXO3) nuclear retention and triggering cell cycle initiation. Notably, while this proinflammatory signaling has been demonstrated in models of diet-induced ovarian inflammation (41), direct evidence under purely physiological conditions remains limited; physiological follicle activation involves tightly regulated and transient inflammatory cues that resolve rapidly; persistent inflammation would be detrimental to follicle integrity (43, 44). Concomitantly, macrophage-derived growth factors including epidermal growth factor (EGF), insulin-like growth factor (IGF), hepatocyte growth factor (HGF), and vascular endothelial growth factor (VEGF) stimulate pre-granulosa cell proliferation and angiogenesis, providing structural and metabolic support for the nascent follicle (40).

Importantly, this activation process involves a coordinated shift in macrophage functional state. Cells with pro-inflammatory characteristics initiate the activation signal, while cells with reparative characteristics subsequently secrete Interleukin-10 (IL-10) and transforming growth factor-beta (TGF-β) to resolve inflammation, remodel extracellular matrix, and maintain vascular stability (22, 42). These states are operational descriptors of functional trends rather than discrete, stable subsets. The balance between these signals determines the amplitude and duration of follicle activation, and its perturbation can lead to either premature depletion or pathological retention of the ovarian reserve.

Macrophage-derived EVs have emerged as important mediators of this regulation. Vesicles from reparative macrophages can enhance PI3K/Akt signaling and promote primordial follicle recruitment in aged ovaries (42). Whether this reflects net preservation or accelerated utilization of the reserve remains unresolved, because increased recruitment could deplete the pool more rapidly unless accompanied by enhanced follicle survival or reduced atresia. This observation therefore indicates a modulatory role in follicle activation rather than a demonstrated reserve-sparing effect, and also illustrates the pathway’s functional plasticity, as PI3K/AKT/mTOR activation in this context is not restricted to pro-inflammatory states (**Figure 1**).

### Macrophages in oocyte growth and maturation: direct oocyte endpoints and evidence grading

Direct oocyte endpoints considered here include spindle organization, meiotic progression, mitochondrial function, and developmental competence; indirect ovarian endpoints include theca angiogenesis and granulosa–immune crosstalk. Beyond their role in follicle activation, macrophages also exert regulatory influences during oocyte growth and maturation. As oocytes progress through the primary and secondary stages of follicular development—stages during which immune cell density in the theca-stromal compartment increases notably compared with the primordial follicle pool (32, 34) —macrophages localize in the theca cell layer, where they support the angiogenesis required to supply the growing follicle with nutrients and oxygen (13, 51). Crucially, even as the blood-follicle barrier establishes immune privilege around the preantral follicle, granulosa cells serve as active intermediaries in immune-ovarian communication. They produce chemokines including CCL2 and CXCL12 that recruit macrophages and T cells to the perifollicular stroma (13, 40), and they express receptors for inflammatory cytokines such as TNF-α and IL-6, enabling them to transduce immune signals to the developing oocyte (46). This granulosa-immune crosstalk represents a key pathway through which the systemic immune milieu influences oocyte quality without direct immune cell access to the oocyte itself. This vascular support is important for oocyte quality, as hypoxia or nutrient insufficiency during follicle growth has been associated with compromised meiotic competence and developmental potential in preclinical models (51).

Macrophages also influence oocyte maturation through paracrine signaling (52). The cytokines they produce, including TNF-α and IL-6, can affect oocyte spindle structure and meiotic progression. Elevated TNF-α levels have been shown in preclinical studies to disrupt metaphase II spindle organization in mouse and porcine oocytes (53). These findings suggest that cytokines essential for normal follicle activation may impair oocyte quality when present at excessive levels or at inappropriate times; direct evidence in human oocytes is lacking.

### Dendritic cells in ovulatory inflammation and immune regulation

Dendritic cells (DCs) are present in the ovary at all stages of follicular development. Unlike macrophages, which are primarily tissue-resident, DCs may be recruited to the ovary in a gonadotropin-dependent manner (54). Their functions extend beyond classical antigen presentation to include the regulation of follicle growth and ovulation.

In the preovulatory follicle, DCs contribute to the inflammatory cascade that culminates in follicle rupture. They express CD11c and possess anti-inflammatory capabilities that help limit the extent of ovulation-associated inflammation (54). This regulatory function is important because the inflammatory response to ovulation, if unchecked, could damage the oocyte and surrounding tissues. DCs also produce factors that promote follicle expansion and corpus luteum formation, further integrating immune and reproductive processes.

### Ovulation-associated inflammation

Ovulation is a regulated sterile inflammatory process, characterized by the recruitment and activation of immune cells within the preovulatory follicle, leading to follicular rupture and oocyte release (49, 55). This inflammatory cascade is initiated by the luteinizing hormone (LH) surge, which induces the expression of inflammatory mediators—including IL-1β, TNF-α, IL-6, and prostaglandins (particularly PGE_2_ and PGF_2_α)—by granulosa cells, theca cells, and infiltrating immune cells (56, 57). These signals promote the expression of matrix metalloproteinases (MMPs) and other proteolytic enzymes that degrade the follicular wall, enabling oocyte expulsion (58).

Macrophages accumulate in the theca layer of the preovulatory follicle and are essential for the ovulatory process. Depletion of ovarian macrophages in animal models disrupts ovulation, underscoring their non-redundant role (48, 51). These macrophages produce both pro-inflammatory cytokines that drive follicular rupture and reparative factors that initiate post-ovulatory tissue remodeling (13, 46). Mast cells, which are present in the ovarian stroma, also contribute to ovulation through the release of histamine and proteases that promote vasodilation, vascular permeability, and matrix degradation (59). Neutrophils are among the first immune cells recruited to the ovulatory site, and their release of proteases and reactive oxygen species (ROS) facilitates the tissue breakdown required for follicle rupture (49). However, excessive or prolonged neutrophilic inflammation may contribute to elevated oxidative stress in the follicular microenvironment, which has been shown to compromise oocyte meiotic competence and developmental potential through ROS-mediated spindle disruption and chromosomal misalignment (49, 53, 60).

The ovulatory inflammatory response must therefore be temporally controlled and self-limiting. Successful ovulation requires a rapid resolution of inflammation following follicular rupture, mediated in part by the clearance of apoptotic cells and the polarization of macrophages toward reparative (M2-like) phenotypes that secrete IL-10 and TGF-β (40). Incomplete resolution of ovulatory inflammation—whether due to chronic low-grade inflammation, impaired macrophage function, or dysregulated cytokine signaling—may create a prolonged inflammatory milieu that has been associated, primarily in preclinical models, with impaired oocyte mitochondrial function, spindle integrity, and fertilization capacity. Indeed, sustained TNF-α exposure directly disrupts metaphase II spindle organization and chromosome alignment in mouse oocytes (61), and systemic inflammatory states have been associated with impaired oocyte developmental competence (62). These findings support a mechanistic link between dysregulated inflammation and oocyte quality in preclinical models, with human evidence remaining correlative. Moreover, this connection is particularly relevant in the context of ovarian aging, where the progressive dysregulation of immune function (inflammaging) may impair the normal ovulatory inflammatory response, contributing to the age-related decline in oocyte quality (63, 64).

### T lymphocytes: balancing immune surveillance and tolerance with consequences for oocyte competence

T-cell effects on the oocyte are primarily indirect, mediated through granulosa cells and follicular fluid cytokines; direct oocyte endpoints are inferred mainly from preclinical models and human correlative data. The adaptive immune system contributes to ovarian homeostasis through the activities of T lymphocytes. T cells are present in the ovary throughout reproductive life, with distinct spatial distributions and functional states (34). In the healthy ovary, CD4+ T helper cells and CD8+ cytotoxic T cells are distributed throughout the stromal compartment, with preferential accumulation in the theca layers of growing follicles and in the corpus luteum (32, 34). Their functions extend beyond classical immune surveillance to include active participation in ovarian remodeling: CD4+ T cells secrete cytokines that modulate steroidogenesis and granulosa cell function, while CD8+ T cells contribute to the elimination of apoptotic cells during follicular atresia and luteal regression (49, 65).

Regulatory T cells (Tregs) are critical for maintaining immune tolerance in the ovary (34, 66). In primary POI patients, Treg deficiency has been associated with a Th1 shift, granulosa cell apoptosis, impaired steroidogenesis, and follicle loss (67); these human data are correlative and do not establish causality. Beyond Tregs, the balance among CD4+ effector subsets—Th1, Th2, and Th17—influences the ovarian inflammatory milieu. Th1 cells produce IFN-γ, which can promote ovulation and luteal function when appropriately regulated, but excessive Th1 activity is associated with follicular damage and poor oocyte quality (67). Conversely, Th2 cytokines such as IL-4 and IL-13 are implicated in tissue repair and vascular remodeling during corpus luteum formation (35). Single-cell transcriptomic profiling of peripheral blood from primary POI patients has further corroborated this immunosenescent phenotype, revealing expanded effector memory T-cell populations and elevated inflammatory signatures in myeloid compartments (68). Treg/Th17 imbalances have been reported as correlative findings in peripheral blood (69) and endometrium (19), but their intraovarian contribution and causal relationship to oocyte quality remain incompletely defined. **Figure 2** synthesizes these correlative human data with preclinical mechanistic insights as a conceptual framework to guide future investigation.

**Figure 2:**
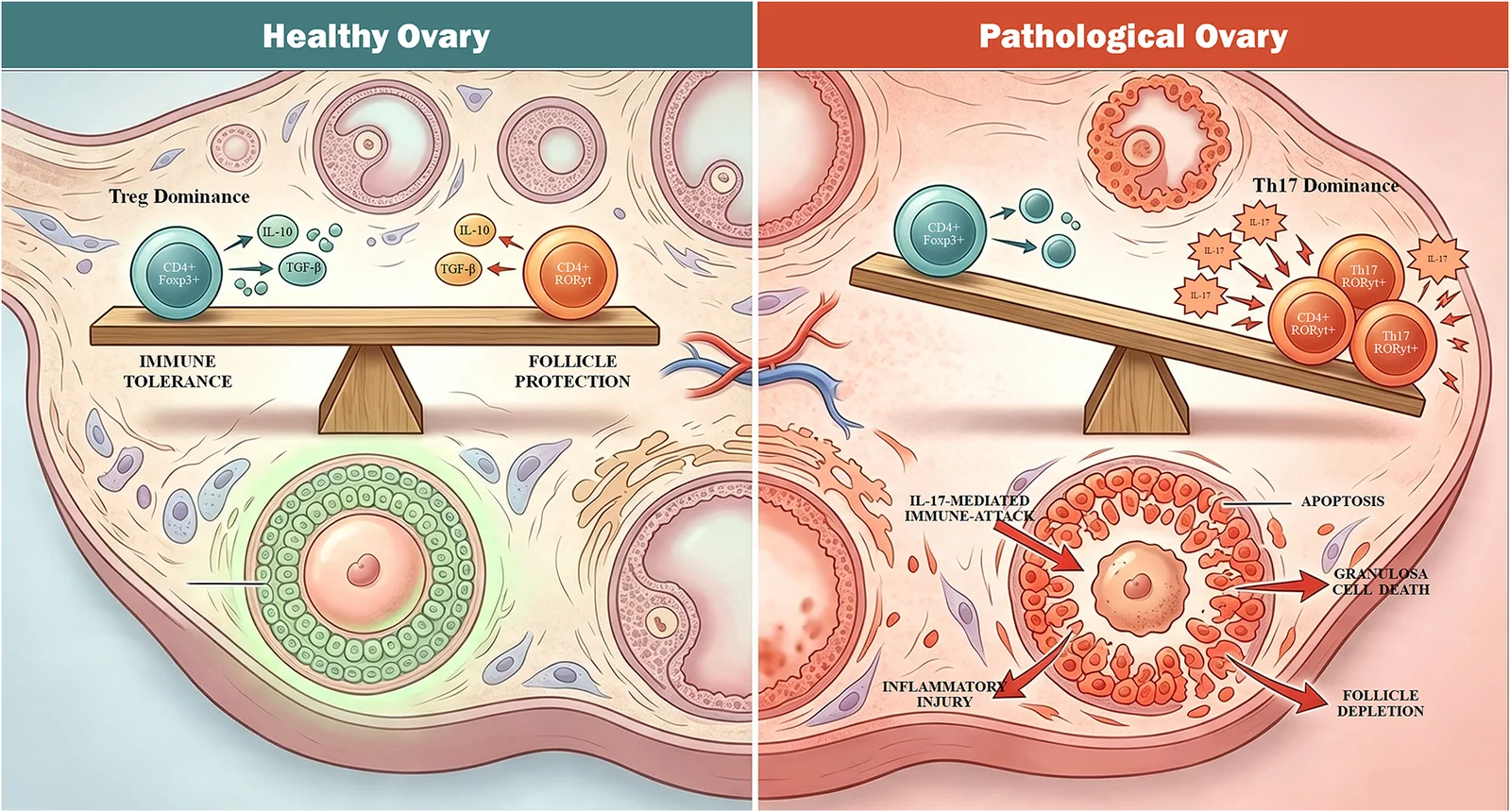
Conceptual model of CD4+ T cell balance in the ovarian microenvironment. Using a simplified model, the diagram contrasts CD4+ T cell balance and its downstream effects on ovarian follicles. The human evidence underlying this model is derived primarily from studies of primary POI, in which Treg deficiency has been associated with a Th1 response and granulosa cell dysfunction; direct intraovarian validation of a Treg/Th17 mechanism remains limited. *(Left) Healthy Ovarian:* The microenvironment is characterized by a state of Treg dominance, where CD4+ Foxp3+ regulatory T cells (Tregs) secrete immunosuppressive cytokines (IL-10 and TGF-β). This cytokine profile maintains immune tolerance, counterbalances the activity of CD4+ RORγt+ cells, and provides protection to ovarian follicles, preserving normal follicular architecture. *(Right) Pathological Ovarian:* In primary POI, Treg deficiency has been associated with a Th1 response, leading to granulosa cell apoptosis, impaired steroidogenesis, and follicle loss. Expansion of CD4+ RORγt+ Th17 cells and excessive IL-17 secretion are depicted as a plausible extension of this model, but have not been directly demonstrated within the human ovary. The resulting follicular destruction shown here represents a conceptual extrapolation requiring direct ovarian validation. Treg, regulatory T cell; Th17, T helper 17 cell; IL, interleukin; TGF-β, transforming growth factor-beta.

### Immune checkpoints in ovarian function: follicle reserve endpoints and uncertain oocyte effects

The direct oocyte endpoint—meiotic competence and developmental potential—has not been systematically assessed in human ICI-exposed oocytes; current evidence is limited to ovarian reserve and follicular endpoints. Immune checkpoints, inhibitory receptors that regulate T cell activation, are increasingly recognized as important players in ovarian physiology. Programmed cell death protein 1 (PD-1), programmed death-ligand 1 (PD-L1), and cytotoxic T-lymphocyte-associated protein 4 (CTLA-4) are expressed within ovarian tissue, including in oocytes, granulosa cells, and stromal compartments (21, 66, 70). Granulosa cells, in particular, may utilize PD-L1 expression to suppress local T-cell activity, thereby helping to maintain the immune-privileged status of the developing follicle (66). They likely contribute to the maintenance of immune tolerance and the prevention of excessive inflammation during follicular development (21, 66).

The therapeutic use of immune checkpoint inhibitors (ICIs) for cancer has drawn attention to the potential reproductive consequences of checkpoint blockade. Emerging evidence from preclinical models suggests that ICI treatment can disrupt ovarian function, reducing primordial follicle numbers and impairing ovulation (70, 71). The mechanisms underlying these effects are multifactorial and may involve T cell-mediated attack on ovarian tissues, disruption of local immune tolerance, and off-target effects of checkpoint signaling pathways.

Current evidence suggests that different ICI classes may exert distinct effects on the ovary. Anti-PD-1/PD-L1 agents (e.g., pembrolizumab, nivolumab, atezolizumab) primarily target the PD-1/PD-L1 pathway, which plays a critical role in maintaining ovarian immune privilege through PD-L1 expression on granulosa cells and oocytes (66). Preclinical studies demonstrate that PD-L1 blockade increases intraovarian effector memory T cells, disrupts follicle development, and induces extrinsic apoptosis via caspase-8 activation (70). By contrast, anti-CTLA-4 agents (e.g., ipilimumab) target a distinct checkpoint predominantly expressed on T cells, where CTLA-4 regulates early T-cell activation and Treg function (66). The two pathways differ mechanistically: PD-1/PD-L1 blockade primarily disrupts local immune tolerance at the tissue level, while CTLA-4 blockade may perturb Treg-mediated suppression, potentially unleashing autoreactive T cells against ovarian antigens (70). Preclinical models indicate that dual PD-1/CTLA-4 blockade produces more profound ovarian damage than either agent alone, suggesting additive or synergistic effects (70).

Notably, a discordance exists between preclinical and clinical evidence. While animal models consistently demonstrate ICI-induced primordial follicle depletion and ovulation defects, available clinical data do not demonstrate clear additional gonadotoxicity associated with ICIs beyond that of conventional chemotherapy (71). Clinical cohorts have not shown consistent suppression of anti-Müllerian hormone or follicle-stimulating hormone following pembrolizumab addition to chemotherapy. This discrepancy may reflect species-specific differences in ovarian physiology, the relatively short duration of clinical follow-up, or confounding by tumor-related systemic inflammation (71). Furthermore, ICIs can indirectly impair ovarian function through immune-related adverse events affecting the hypothalamic-pituitary-gonadal axis, particularly hypophysitis and thyroiditis, which may cause hypogonadotropic ovarian insufficiency secondary to endocrine dysfunction. This entity is a form of secondary ovarian insufficiency and is distinct from primary POI, which denotes primary ovarian failure; the two should not be conflated.

These observations highlight that ICI effects on the ovary are target-dependent, context-specific, and mediated through both direct immune activation within ovarian tissue and indirect endocrine disruption. Prospective studies with standardized fertility endpoints are needed in women of reproductive age receiving ICI therapy.

### NK cells and neutrophils: indirect ovarian endpoints and potential oocyte consequences

Natural killer (NK) cells, a subset of innate lymphoid cells, are present in the ovary and contribute to angiogenesis and tissue remodeling through the release of VEGF and interferon-gamma (IFN-γ) (72, 73). Their roles in ovulation and corpus luteum function are areas of active investigation.

The corpus luteum contains a high density of immune cells. Macrophages are abundant in the developing and regressing corpus luteum and contribute to luteal angiogenesis, progesterone production, and the clearance of apoptotic luteal cells during luteolysis (40, 51). Natural killer (NK) cells, which are enriched in follicular fluid and secrete IFN-γ in the ovarian microenvironment (72), may contribute to luteal vascular remodeling through cytokine-mediated pathways in concert with other angiogenic factors. Direct evidence for NK cell-mediated VEGF production within the human corpus luteum, however, remains limited and is primarily derived from studies of decidual NK cells and other reproductive tissues (74). Dysregulation of luteal immune homeostasis—whether due to inadequate macrophage recruitment, impaired NK cell function, or aberrant cytokine signaling—may compromise luteal progesterone production and structural integrity, with potential carryover effects on subsequent follicular development and oocyte quality in successive cycles (21, 22).

Neutrophils, though less abundant in the healthy ovary, are among the first immune cells to respond to ovarian stress. They can be recruited to the ovary following injury or inflammation and may contribute to follicle damage through the release of proteases and reactive oxygen species (ROS). Conversely, in controlled contexts, neutrophil activity may facilitate the tissue remodeling required for follicle rupture during ovulation (49). **Table 2** summarizes the key immune cell populations in the ovarian microenvironment, their distribution, physiological functions, and dysregulation-associated changes during aging.

**Table 2:** Ovarian microenvironment and roles in oocyte competence of immune cells.

Immune cell type	Distribution	Functions in ovarian physiology	Dysregulation/aging-associated changes	References
Macrophages	Abundant throughout ovary; enriched in theca cell layers of growing follicles and corpus luteum.	Key modulators: M1-like promote follicle activation and transient inflammation (preclinical); M2-like mediate resolution, vascular integrity, and ECM remodeling; EVs enhance PI3K/Akt signaling.	Aged ovaries show reduced M2-like populations and increased M1/M2 ratio. Impaired tissue repair and amplified proinflammatory signaling. Altered EV cargo may impair rather than promote oocyte quality.	(13, 14, 22, 40–45, 51–53, 63, 64, 78, 79)
Dendritic Cells (DCs)	Present at all follicular stages; recruited in gonadotropin-dependent manner.	Contribute to the inflammatory cascade for follicle rupture. Express CD11c and exert anti-inflammatory functions to limit ovulation-associated inflammation. Promote follicle expansion and corpus luteum formation.	Not explicitly detailed in aging; generally linked to regulation of ovulation-associated inflammation.	(54)
T Lymphocytes	Present throughout reproductive life; distinct spatial distributions.	Tregs: Maintain immune tolerance; suppress excessive inflammation; prevent autoimmune attack. Treg/Th17 balance: Critical for ovarian homeostasis; imbalance linked to granulosa cell apoptosis and follicle loss.	Treg/Th1 shift in primary POI (human ovarian tissue/peripheral blood); Treg/Th17 imbalance in unexplained infertility (human peripheral blood) and in repeated implantation failure (human endometrium). These findings derive from distinct biological compartments and should not be conflated as a single ovarian microenvironment phenomenon; direct ovarian validation remains lacking. Treg deficiency recapitulates primary POI-like phenotypes (Th1 shift, impaired steroidogenesis).	(19, 66, 67, 69)
Natural Killer (NK) Cells	Present in ovary; enriched in follicular fluid.	Contribute to angiogenesis and tissue remodeling via VEGF and IFN-γ. Roles in ovulation and corpus luteum function under investigation.	Not explicitly detailed in aging; generally associated with vascular and tissue remodeling.	(72, 73)
Neutrophils	Less abundant in healthy ovary; recruited upon injury/inflammation.	Facilitate tissue remodeling for follicle rupture during ovulation. Can cause follicle damage via proteases and ROS in pathological contexts.	Not explicitly detailed in aging; implicated in acute inflammatory responses.	(49)

Functions listed are derived from human descriptive studies, animal models, or both, as indicated by the cited references. Direct oocyte endpoints are not implied unless explicitly stated. Dysregulation-associated changes are largely derived from preclinical models.

## Immune mechanisms affecting oocyte meiotic competence and chromosomal integrity

Beyond their roles in follicle growth and somatic support, immune-derived signals directly influence the meiotic machinery of the oocyte. Chromosome segregation during meiosis I and II is sensitive to the inflammatory microenvironment.

Tumor necrosis factor-alpha (TNF-α) has been shown to disrupt metaphase II spindle organization in mouse oocytes, increasing the incidence of chromosome misalignment and aneuploidy (52). Similarly, interleukin-6 (IL-6) exposure is associated with spindle abnormalities and impaired polar body extrusion (53). These effects are mediated in part through oxidative stress pathways: inflammatory cytokines stimulate the production of reactive oxygen species (ROS) within the oocyte, which in turn damage spindle microtubules and kinetochore function (60).

The NLRP3 inflammasome, activated by mitochondrial ROS in aging oocytes, amplifies this inflammatory cascade through IL-1β production, creating a feedback loop that further compromises meiotic fidelity (75, 76). Notably, the meiotic spindle appears to be particularly vulnerable during the prolonged arrest at metaphase II, a period during which oocytes are exposed to cumulative inflammatory insults.

While animal model data provide mechanistic insight, direct evidence linking specific immune signals to aneuploidy in human oocytes remains limited. Most human studies are correlative, associating follicular fluid cytokine levels with oocyte maturation rates rather than directly quantifying chromosomal abnormalities. Future studies are needed to directly assess immune-aneuploidy relationships in human oocytes.

## Immunological mechanisms of age-related oocyte quality decline

### Inflammaging and the ovarian immune microenvironment: direct oocyte endpoints and indirect ovarian endpoints

Direct oocyte endpoints reported in preclinical models include spindle disruption, oxidative DNA damage, and impaired polar body extrusion; indirect ovarian endpoints include fibrosis, vascular degeneration, and altered granulosa–oocyte communication. Evidence is predominantly preclinical, with human correlative support from follicular fluid cytokine studies. Ovarian aging is characterized by a state of chronic, low-grade inflammation termed inflammaging (63, 64). Spatial transcriptomic and single-cell profiling have recently dissected the multicellular origins of this process in the murine ovary, identifying specific pro-inflammatory macrophage and T-lymphocyte subpopulations that expand with age and engage in bidirectional signaling with granulosa cells through TNF, CXCL, and TGFβ pathways (63). Notably, the overall immune cell population in the aged ovary is significantly expanded compared with young counterparts, with macrophages, T cells, and other leukocyte subsets showing increased abundance within the stromal and perifollicular compartments (63). This framework integrates inflammation, immune dynamics, and senescence as interrelated processes in ovarian aging (23). Complementary single-cell analyses have further revealed that ovarian myeloid cells exhibit divergent inflammatory outputs beyond the classical M1/M2 dichotomy (24). This reflects age-related immune dysregulation, manifesting as elevated proinflammatory cytokines and impaired resolution of inflammation.

In the aging ovary, inflammaging is driven by multiple mechanisms: the senescence-associated secretory phenotype (SASP) of accumulated senescent cells, mitochondrial dysfunction, and the progressive loss of regulatory immune populations (77, 78). Proinflammatory cytokines, including IL-6 and TNF-α, accumulate in the ovarian microenvironment with age; their effects on the oocyte spindle and oxidative stress pathways are described above. Direct evidence in human oocytes remains limited (62).

### Macrophage dysfunction in the aging ovary: indirect ovarian endpoints with inferred oocyte consequences

Direct oocyte endpoints are inferred rather than measured; the primary evidence concerns macrophage polarization, angiogenesis, and cytokine milieu, and is largely derived from animal models. Aging is associated with shifts in macrophage phenotype and function within the ovary. Single-cell transcriptomic analyses have revealed that aged ovaries exhibit reduced M2-like populations and an increased ratio of proinflammatory macrophages (63, 79). This shift diminishes the ovary’s capacity for tissue repair and immune regulation while amplifying inflammatory signaling.

The functional consequences of macrophage dysregulation are manifold. Reduced M2 macrophage activity impairs angiogenesis, compromising the vascular support essential for follicle development. Increased proinflammatory cytokine production creates a hostile environment for oocyte development, promoting oxidative stress and meiotic dysfunction (53, 61). Macrophage-derived EVs, which in young ovaries support follicular activation, may acquire altered cargo in aging that impairs rather than promotes oocyte quality.

Single-cell analysis of ovarian myeloid cells has identified five macrophage subsets, including a Cx3cr1-low/Cd81-high subset unique to aged murine ovaries (79). This indicates that age-restricted macrophage states can be defined at the transcriptional level rather than only inferred from quantitative shifts. While current single-cell studies have primarily identified quantitative shifts and compositional changes in existing immune populations with age (24, 63, 79), these findings suggest that age-restricted subsets may serve as candidate therapeutic targets or biomarkers of ovarian aging. Future work should determine whether analogous subsets exist in the human ovary and whether they represent causal drivers or correlates of ovarian aging.

### The NLRP3 inflammasome in oocyte aging: direct oocyte endpoints and preclinical therapeutic evidence

In mouse models, direct oocyte endpoints include meiotic spindle disruption, mitochondrial membrane potential collapse, increased oocyte ROS, and reduced blastocyst formation; human evidence remains correlative. **Figure 3** depicts the mitochondria-driven NLRP3 inflammasome axis and its pathological feedback loop, showing how mitochondrial ROS trigger inflammasome assembly and subsequent IL-1β production, which in turn exacerbates mitochondrial dysfunction.

**Figure 3:**
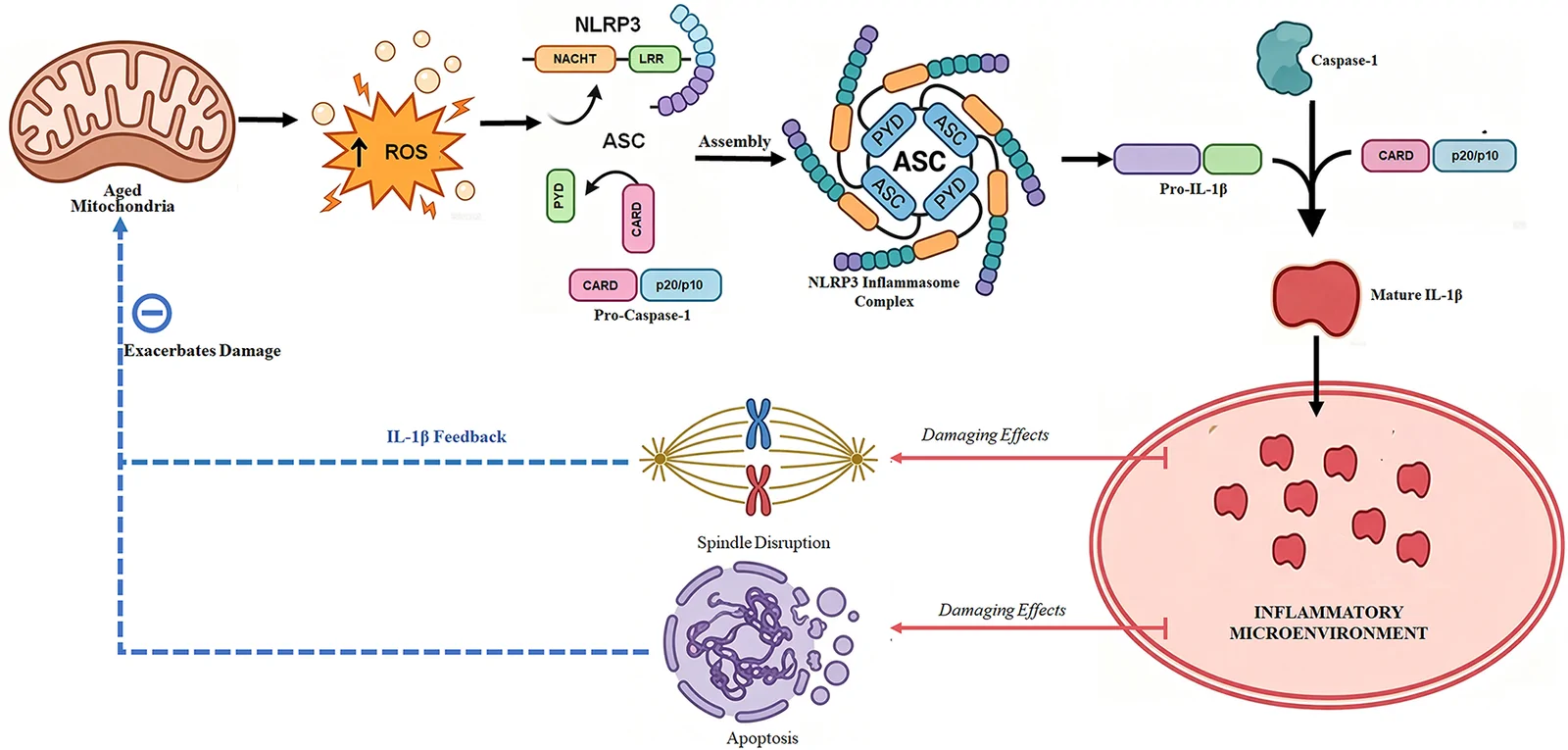
Mitochondria-driven NLRP3 inflammasome axis and its pathological feedback loop. Aged mitochondria generate excessive reactive oxygen species (ROS), which serve as a trigger for the assembly of the NLRP3 inflammasome complex. This complex comprises NLRP3, ASC, and Pro-Caspase-1, leading to the activation of Caspase-1. Active Caspase-1 subsequently cleaves Pro-IL-1β into its mature form, Mature IL-1β. The release of Mature IL-1β establishes a robust inflammatory microenvironment, which exerts damaging effects on cellular structures, specifically inducing spindle disruption during meiosis and promoting apoptosis. This cascade forms a positive feedback loop: cellular damage caused by spindle disruption and apoptosis further exacerbates mitochondrial dysfunction, which amplifies ROS production and thereby sustains inflammasome activation. This model is based primarily on preclinical evidence; direct validation in human oocytes is lacking. ROS, reactive oxygen species; IL-1β, interleukin-1 beta; ASC, apoptosis-associated speck-like protein containing a CARD.

The NLRP3 inflammasome is a mediator of innate immune responses and has been implicated in ovarian aging. This multiprotein complex, when activated by oxidative stress or other danger signals, promotes the production of IL-1β and interleukin-18 (IL-18), which can damage ovarian tissues (75, 76). NLRP3 activation has been associated with mitochondrial dysfunction in mouse models of ovarian aging; human data remain correlative. Aging oocytes accumulate damaged mitochondria that produce excess ROS, triggering NLRP3 inflammasome activation (76, 80). The resulting IL-1β production contributes to the inflammatory microenvironment that impairs oocyte quality (75, 76, 81). Pharmacological inhibition of NLRP3 has been shown to prevent ovarian aging in mouse models, suggesting that targeting this pathway represents an experimental approach requiring further validation in preclinical models (80, 82, 83). However, these findings remain at the preclinical stage; human evidence is currently limited to correlative expression and association studies rather than direct demonstrations of therapeutic efficacy. Clinical translation will require rigorous evaluation of safety, dosing, and fertility outcomes in human subjects.

### Mitochondrial dysfunction and immune-reproductive axis disruption: oocyte-intrinsic vulnerability and compartmental asymmetry

This section focuses on why the oocyte, unlike granulosa cells, accumulates mitochondrial damage and how this asymmetry shapes direct oocyte endpoints. Mitochondria are central to both oocyte quality and immune function. In oocytes, mitochondrial integrity is essential for energy production, calcium buffering, and the regulation of apoptosis. In immune cells, mitochondria regulate activation, cytokine production, and metabolic reprogramming.

Mitochondrial quality control differs fundamentally between the oocyte and its surrounding somatic cells, and this asymmetry has direct implications for how mitochondrial decline is interpreted across follicle compartments. Granulosa cells and cumulus cells possess robust mitophagy machinery and can efficiently clear damaged mitochondria through PINK1/Parkin-dependent and receptor-mediated pathways, thereby maintaining a relatively healthy mitochondrial pool throughout follicular development (84, 86). The oocyte, by contrast, is a largely post-mitotic cell with a finite, non-renewable mitochondrial complement that is established during oogenesis and is not replenished thereafter. Even in young, healthy oocytes, mitophagy is inefficient: the oocyte actively suppresses canonical autophagic flux during meiotic maturation to avoid inappropriate degradation of maternal stores, and its capacity to selectively eliminate dysfunctional mitochondria is limited compared with granulosa cells (78, 84). Consequently, damaged mitochondria accumulate in the oocyte with each developmental insult, and this vulnerability is intrinsic to the oocyte rather than being solely a consequence of aging.

This compartmental asymmetry means that mitochondrial dysfunction in the follicle is not a uniform process. Granulosa cells may transiently exhibit mitochondrial stress and elevated ROS yet recover through effective mitophagy, whereas the oocyte exposed to the same microenvironment retains damaged organelles. The oocyte therefore functions as a sink for mitochondrial damage generated across the follicle, including damage originating in granulosa cells and immune cells. This asymmetry also shapes the directionality of immune-metabolic crosstalk: ROS and mitochondrial DNA released from granulosa cells or immune cells can be transferred to or sensed by the oocyte, where they trigger NLRP3 inflammasome activation and cGAS-STING signaling, while the oocyte’s own impaired mitophagy prevents resolution of the underlying organelle damage (78, 82, 95).

Aging is associated with mitochondrial dysfunction in both compartments, creating a self-reinforcing cycle that accelerates reproductive decline (82, 84). Because the oocyte cannot compensate through mitophagy as granulosa cells do, age-related mitochondrial decline in the oocyte is effectively cumulative and irreversible, whereas granulosa cell mitochondrial function may be partially preserved or restored. In preclinical models, mitochondrial dysfunction in both compartments promotes proinflammatory phenotypes and reduces oocyte developmental competence through NLRP3 activation; human evidence remains correlative. The mitochondria-driven inflammation that results has been proposed as a new frontier in understanding ovarian aging (76).

### The SASP and ovarian immune senescence: indirect ovarian endpoints and granulosa–oocyte communication

In preclinical models, direct oocyte endpoints include disrupted gap junction–mediated meiotic arrest and inflammatory cytokine–induced spindle damage; indirect endpoints include fibrosis and vascular degeneration. Human evidence is largely correlative. Cellular senescence is defined as irreversible cell cycle arrest with resistance to apoptosis and secretion of pro-inflammatory SASP factors (85, 86). Senescent cells accumulate in the ovary with age, beginning as early as 9–12 months in mice and correlating with declining ovarian reserve after age 35 in humans (63). This process is associated with follicle loss and inflammaging: senescent granulosa cells induce apoptosis in neighboring cells via SASP, while SASP-driven proinflammatory polarization of macrophages creates chronic inflammation that in turn promotes further senescence, forming a self-reinforcing cycle (85, 86). The downstream amplification of this cycle through NLRP3 inflammasome activation is discussed in the preceding section.

The consequences of SASP-mediated inflammation for oocyte quality are direct and indirect. Direct effects include the disruption of oocyte meiotic machinery by inflammatory cytokines. Indirect effects include the promotion of ovarian fibrosis, which impairs follicle development and oocyte maturation, and the formation of a microenvironment that impairs oocyte survival (86, 87). SASP-mediated inflammation also promotes vascular degeneration, characterized by thickened vessels and reduced ovarian blood flow, which creates a hypoxic microenvironment that further exacerbates M1 macrophage polarization and SASP release, establishing a self-perpetuating cycle of tissue damage.

SASP factors may also directly impair granulosa-oocyte communication by disrupting gap junctions. TNF-α downregulates connexin-43 (Cx43) expression in granulosa cells (88), compromising the transfer of cAMP and cGMP required for oocyte meiotic arrest (89). This disruption represents an additional mechanism through which the SASP-enriched microenvironment compromises oocyte developmental competence.

**Figure 4** presents the SASP-driven pathological cascade and feedback loops mediating ovarian aging, depicting how senescent granulosa cells initiate inflammation, fibrosis, and vascular degeneration through self-perpetuating cycles.

**Figure 4:**
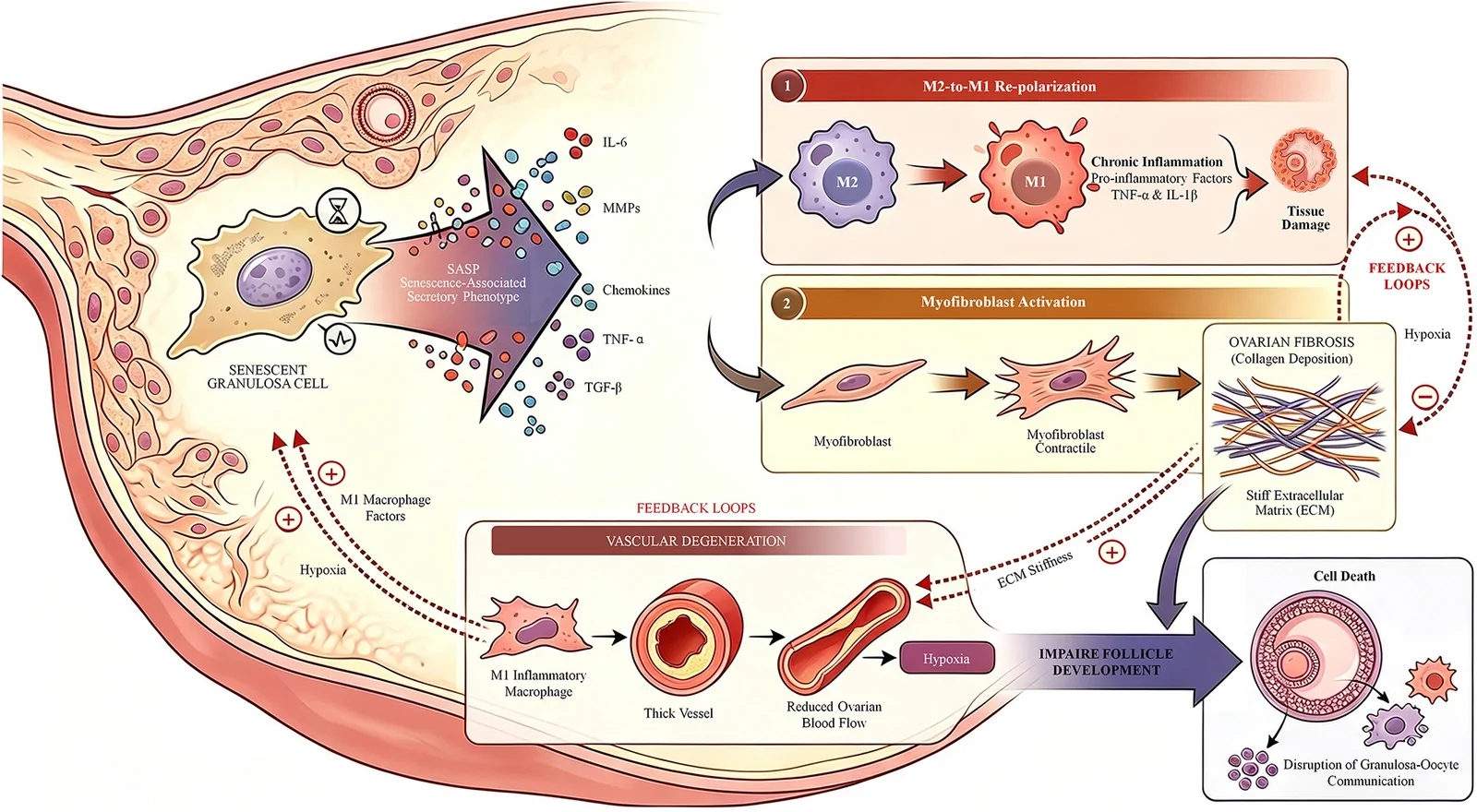
SASP-driven pathological cascade in ovarian aging. Senescent granulosa cells secrete SASP factors (IL-6, TNF-α, TGF-β, MMPs), driving M1 macrophage polarization and myofibroblast activation, which lead to chronic inflammation and fibrosis, respectively. The M1→M2 arrow represents a resolution attempt that fails in the SASP-rich milieu; sustained TNF-α/IL-1β production is driven by continuous M2-to-M1 repolarization, not by M2 macrophages. Inflammation and fibrosis promote vascular degeneration and hypoxia, which feedback to amplify M1 polarization and SASP release, forming self-perpetuating cycles that impair follicular development and granulosa-oocyte communication. This cascade is supported mainly by preclinical studies; human evidence remains correlative.

### Iron metabolism and ferroptosis: direct oocyte endpoints

In preclinical models, direct oocyte endpoints include lipid peroxidation–induced membrane damage, reduced maturation and fertilization rates, and oocyte degeneration; human evidence is emerging but still limited. Recent research has identified ferroptosis, an iron-dependent form of regulated cell death, as a potential contributor to ovarian aging. Ferroptosis is characterized by the accumulation of lipid peroxides in the presence of iron, leading to oxidative membrane damage (4, 90–92). In the immune context, ferroptosis has been implicated in the loss of regulatory immune populations in preclinical models (90); this represents a hypothesis requiring validation in human ovarian tissue. Such loss could potentially exacerbate inflammation and immune dysregulation. The relationship between ferroptosis and oocyte quality is an emerging area of investigation, with implications for understanding how immune-ovarian interactions influence reproductive aging.

### Mitochondrial DNA leakage and innate immune sensing in oocyte aging

Ferroptosis and mitochondrial innate immune sensing are mechanistically linked by mitochondrial damage but represent distinct pathways and are therefore discussed separately. Notably, a recent preclinical study demonstrated that aged oocytes accumulate cytosolic mitochondrial DNA (mtDNA), activate cGAS-STING signaling within the oocyte, and transmit oocyte-derived cGAMP through gap junctions to surrounding granulosa cells; genetic or pharmacological interruption of this pathway ameliorated ovarian dysfunction in mice (93). These findings establish an oocyte-intrinsic innate immune sensing mechanism with intercellular transmission to granulosa cells. However, direct evidence in human aging oocytes remains limited; the cited findings are primarily derived from murine oocyte models and granulosa-cell studies (78). This oocyte-intrinsic cGAS-STING activation may intersect with ferroptosis, because mitochondrial damage can drive both mtDNA release and iron-dependent lipid peroxidation (93). In addition, DAMPs released from dying oocytes are recognized by TLR4 on macrophages, triggering NF-κB signaling and proinflammatory cytokine production, which may in turn exacerbate mitochondrial dysfunction and ferroptosis in neighboring oocytes (90, 93). However, while each of these links is individually plausible, the complete ferroptosis–DAMP–TLR4–NF-κB–ferroptosis loop has not been demonstrated as a single integrated ovarian mechanism.

**Figure 5** provides a proposed integrative model of the self-reinforcing cycle orchestrated by mitochondrial dysfunction, ferroptosis, and macrophage-mediated inflammation in oocyte demise, depicting the crosstalk between ferroptotic oocytes and the innate immune microenvironment through cGAS-STING and TLR4/NF-κB pathways.

**Figure 5:**
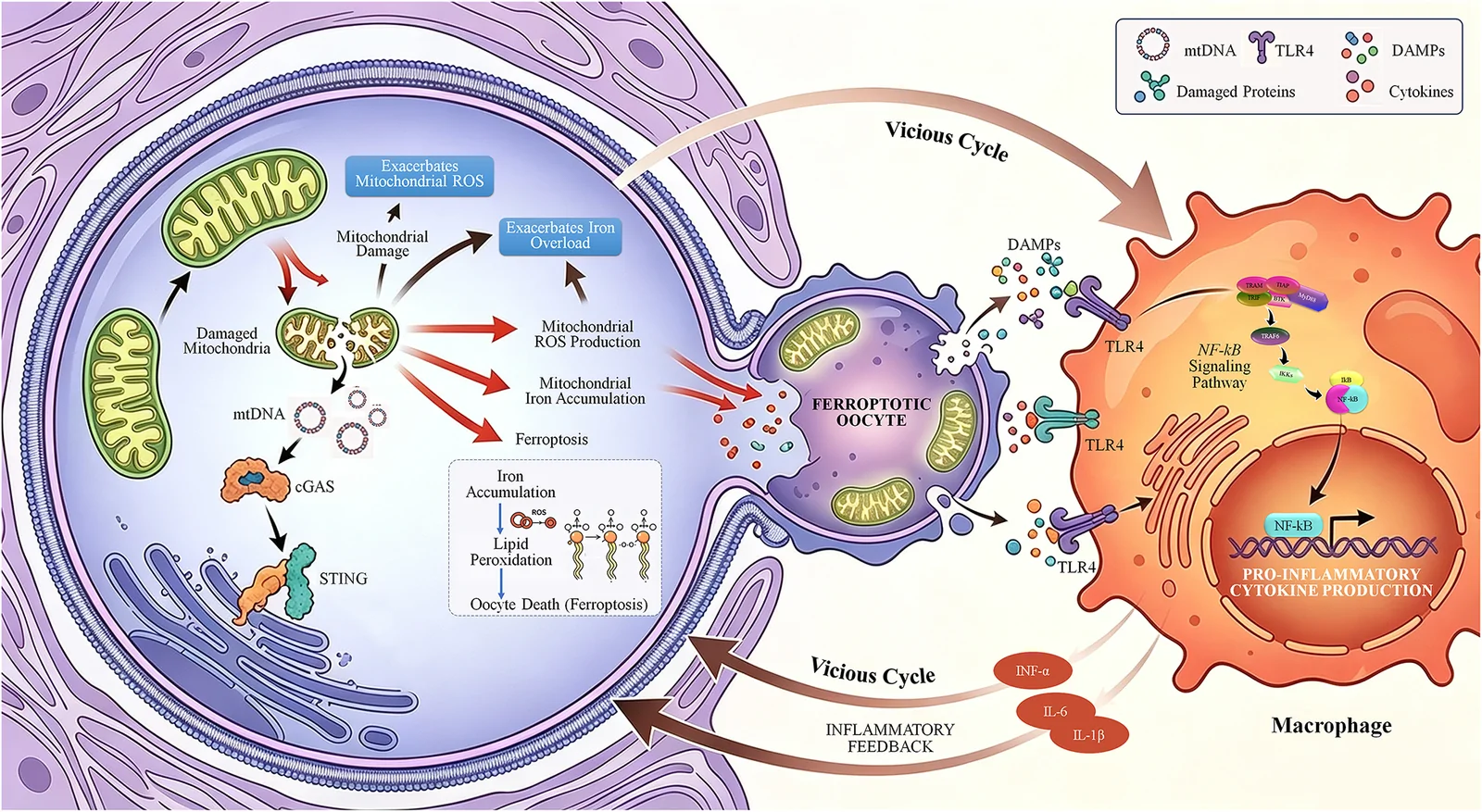
Proposed integrative model of a self-reinforcing cycle orchestrated by mitochondrial dysfunction, ferroptosis, and macrophage-mediated inflammation in oocyte demise. Schematic illustration depicting the molecular crosstalk between a ferroptotic oocyte and the innate immune microenvironment. Within the oocyte, mitochondrial damage serves as a central trigger, driving two parallel pathological cascades:**(i)** the cGAS-STING pathway, activated by cytosolic leakage of mitochondrial DNA (mtDNA), which initiates an intrinsic inflammatory response; and **(ii)** the ferroptotic pathway, fueled by excessive mitochondrial ROS production and iron accumulation, leading to lipid peroxidation and, ultimately, oocyte death. This dying oocyte releases a spectrum of damage-associated molecular patterns (DAMPs), including mtDNA, HMGB1, ATP, oxidized lipids, and heat shock proteins into the extracellular space. These DAMPs are recognized by Toll-like receptor 4 (TLR4) on the surface of infiltrating macrophages, subsequently activating the NF-κB signaling pathway and inducing robust pro-inflammatory cytokine production (e.g., TNF-α, IL-6, IL-1β). Crucially, this macrophage-driven inflammatory response establishes a self-reinforcing cycle (indicated by feedback arrows) by feeding back to the oocyte to exacerbate mitochondrial damage, elevate ROS levels, and intensify iron overload, thereby amplifying the ferroptotic process. This self-perpetuating loop may contribute to mitochondria-immune axis interactions in the pathogenesis of ovarian follicle atresia. This model is presented as a proposed integrative framework rather than an established causal circuit, and is derived largely from murine and granulosa-cell studies; direct evidence in human oocytes remains limited. Cytosolic mtDNA accumulation, oocyte-intrinsic cGAS-STING activation, cGAMP transfer to granulosa cells via gap junctions, and amelioration of ovarian dysfunction upon pathway interruption are supported by direct experimental evidence. However, ferroptosis-mediated DAMP release, TLR4/NF-kB activation in macrophages, cytokine feedback to the oocyte, and iron overload–mediated ferroptosis amplification remain individually plausible but unproven as a single integrated ovarian mechanism.

## Pathological immune dysregulation and oocyte quality decline

### Inflammation and oxidative stress in oocyte quality

The interaction between inflammation and oxidative stress impairs the oocyte developmental environment. Inflammatory cytokines stimulate the production of ROS in both oocytes and their surrounding somatic cells, while oxidative stress triggers inflammatory responses through pathways such as NLRP3 activation (75, 81, 94).

The cumulative effect of this inflammation-oxidative stress cycle has been associated with degradation of oocyte quality through multiple mechanisms, primarily in preclinical models. Oxidative stress damages DNA, proteins, and lipids in oocytes, impairing meiotic competence and developmental potential. Inflammatory cytokines disrupt spindle structure, interfere with chromosome segregation, and promote apoptosis (52, 53, 60). The chronic low-grade inflammation of ovarian aging and the more acute inflammation of autoimmune oophoritis both exploit these pathways.

### Immune dysregulation in primary premature ovarian insufficiency

Primary POI is characterized by the loss of normal ovarian activity before the age of 40 years and affects approximately 3.5% of women globally (95). The etiology of POI is multifactorial, encompassing genetic defects (10-30%) (96), iatrogenic damage (6-47%), autoimmune disorders (5-18%) (97), and environmental exposures, with the majority of cases historically classified as idiopathic (98). Primary POI is a primary ovarian disorder and is distinct from secondary ovarian insufficiency arising from hypothalamic-pituitary dysfunction. The discovery of immune dysregulation in many primary POI patients suggests that POI may in some cases involve early dysregulation of aging-related immune pathways.

The immune signatures of primary POI show similarities to those of physiological ovarian aging in correlative human studies; causal inference requires longitudinal investigation, including elevated proinflammatory cytokines and altered macrophage polarization in ovarian tissue, as well as Treg/Th1 shifts reported in peripheral blood from patients with primary POI (67). Treg/Th17 imbalances have been reported in other infertility contexts (e.g., peripheral blood in unexplained infertility (69), endometrium in repeated implantation failure (19)), but these findings should not be extrapolated to the ovarian microenvironment without direct intra-ovarian validation. Certain immune and senescence-related pathways may overlap between primary POI and physiological ovarian aging, but these conditions are not equivalent biological processes. Genetically mediated POI, iatrogenic POI, autoimmune oophoritis, and physiological reproductive aging differ in etiology, affected cell populations, and temporal dynamics, and should not be interpreted as the same process occurring at different rates (22, 99). These observations suggest that immunomodulatory interventions developed for age-related ovarian decline should not be assumed to be applicable across POI subtypes. Any such application would require subtype-specific evidence, particularly in immune-mediated POI, and early detection of immune dysregulation remains a research direction rather than an established preventive strategy.

### Autoimmunity and the ovary

The ovary is susceptible to autoimmune attack, which can manifest as autoimmune oophoritis and contribute to primary POI. In autoimmune oophoritis, the ovarian cortex is infiltrated by lymphocytes, including T and B cells, and antibodies against ovarian antigens are present (65, 100). The resulting inflammation damages follicles and depletes the ovarian reserve.

The mechanisms that trigger ovarian autoimmunity are not fully understood but likely involve a breakdown of immune tolerance. The expression of immune checkpoint molecules in the ovary suggests that these pathways are important for preventing autoimmune attack, and their dysfunction may contribute to oophoritis. The pregnancy-associated immune adaptations, including the Treg-mediated suppression of maternal immune responses, further underscore the importance of immune tolerance for reproductive health.

## Immunomodulatory strategies for oocyte quality preservation

### Targeting macrophage polarization

The demonstration that macrophage polarization can be pharmacologically modulated has opened therapeutic possibilities for ovarian health. Strategies that promote M2-like (anti-inflammatory/reparative) macrophage phenotypes and limit M1-like (proinflammatory) polarization have shown promise in preclinical models (22, 42). From an oocyte-endpoint perspective, the relevant outcomes are direct oocyte endpoints—spindle integrity, mitochondrial function, developmental competence—and indirect ovarian endpoints—vascular support and cytokine milieu. Evidence remains predominantly preclinical, with oocyte quality usually a secondary endpoint; no human study has yet demonstrated that macrophage polarization improves oocyte competence.

Potential approaches include the administration of IL-10 or other M2-polarizing cytokines, the use of small molecules that inhibit proinflammatory signaling pathways, and the delivery of macrophage-modulating agents via EVs. The safety and efficacy of these approaches in humans remain to be determined, but preclinical evidence suggests that further investigation of macrophage-targeted therapies for improving oocyte quality is warranted.

### Restoring Treg/Th17 balance

The restoration of Treg/Th17 balance has emerged as a promising strategy for preserving ovarian function. In conditions characterized by Treg deficiency or Th17 excess, agents that promote Treg differentiation or function may limit the inflammatory damage to ovarian tissues (22, 101). Direct oocyte endpoints have not been systematically measured for Treg/Th17-targeted interventions; current evidence concerns indirect ovarian endpoints such as granulosa cell apoptosis and follicle loss. Human data remain correlative and largely non-ovarian, and no clinical trial has yet evaluated oocyte competence as an endpoint.

Low-dose IL-2, which selectively expands Treg populations, has been explored in non-ovarian autoimmune diseases and in ovarian cancer immunotherapy (55). Its proposed application to ovarian function in POI or ovarian aging remains a mechanistic hypothesis: Treg expansion has not been evaluated in ovarian tissue, and no clinical study has assessed oocyte competence or fertility outcomes following low-dose IL-2 in this context. Other approaches include the use of metabolic modulators that influence T cell differentiation, given that Treg and Th17 differentiation are regulated by distinct metabolic pathways, but these likewise remain untested in ovarian settings.

### NLRP3 inflammasome inhibition

The central role of NLRP3 inflammasome activation in ovarian aging has made this pathway a candidate target for therapeutic exploration. Small-molecule NLRP3 inhibitors have been shown to prevent ovarian aging in mouse models, with benefits encompassing preservation of follicle reserve (increased primordial and primary follicle counts), protection of oocyte meiotic competence, reduction of IL-1β-driven inflammation and oxidative stress, restoration of mitochondrial function, and attenuation of ovarian fibrosis (75, 76, 80, 82, 83, 86). These findings support NLRP3 inhibition as an experimental strategy requiring clinical validation. However, these findings are exclusively preclinical. Human evidence consists of correlative studies demonstrating NLRP3 expression and IL-1β elevation in ovarian tissues, but no clinical data yet exist to confirm that NLRP3 inhibition preserves fertility or improves oocyte quality in humans. In mouse models, NLRP3 inhibition has been associated with direct oocyte endpoints including preserved meiotic spindle organization, reduced oocyte ROS, and improved blastocyst formation. Human evidence remains correlative, and no clinical study has yet shown improved oocyte quality; NLRP3 inhibition should therefore be regarded as an experimental strategy requiring clinical validation.

The rationale for NLRP3 inhibition is compelling: by blocking the production of IL-1β and IL-18, these agents reduce the inflammatory burden on the ovary without globally suppressing immune function. Nonetheless, clinical translation will require careful evaluation of safety and efficacy, as NLRP3 is also important for host defense against pathogens. At present, NLRP3 inhibition should be viewed as a candidate therapeutic target requiring validation in human studies rather than a clinically established approach for fertility preservation.

## Experimental regenerative and immunomodulatory approaches

### Mesenchymal stem cells and the immune niche

MSCs have immunomodulatory properties that make them attractive for ovarian applications. MSCs can polarize macrophages toward M2 phenotypes, suppress T cell responses, and secrete factors that promote tissue repair (56–58). In preclinical models, MSC administration has been reported to improve ovarian function and oocyte quality in aged or damaged ovaries; these findings support further investigation of MSCs as an experimental approach, though clinical evidence remains limited and preliminary. MSC therapies have most consistently reported indirect ovarian endpoints—follicle reserve, granulosa cell survival, vascular support—whereas direct oocyte endpoints are inconsistently reported and often secondary. Human evidence remains preliminary, and no controlled trial has established an effect on oocyte competence.

### Platelet-rich plasma and ovarian rejuvenation

Platelet-rich plasma (PRP) is not an MSC-based therapy and is discussed separately because its mechanism of action and evidence base differ from those of MSC administration. PRP, which contains high concentrations of growth factors and cytokines, has also been explored for ovarian rejuvenation (102, 103). However, controlled clinical evidence does not support its efficacy. The multicenter PROVA randomized controlled trial found no improvement in metaphase II oocyte yield, blastocyst or euploid blastocyst yield, antral follicle count, anti-Müllerian hormone, or sustained implantation after intraovarian PRP injection in women with poor ovarian response (104). Subsequent randomized data have not been entirely concordant, and the most recent systematic review concludes that uncontrolled studies frequently report improvement whereas controlled and randomized data do not demonstrate consistent improvement in mature-oocyte, pregnancy, or live-birth outcomes. PRP and MSC-based rejuvenation therefore remain experimental and are not validated for routine fertility care. PRP should not be presented primarily as an immunomodulatory “ovarian rejuvenation” strategy; its potential role, if any, requires definition through adequately controlled trials with oocyte competence as a prespecified endpoint. The speculative notion that these approaches might ‘complement or replace’ conventional fertility treatments has been moderated; they should be viewed as potential adjuncts to existing approaches should their safety and efficacy be validated in controlled clinical trials.

### Extracellular vesicles as therapeutic agents

EVs derived from macrophages or MSCs carry microRNAs, proteins, and lipids that can modulate immune cell function and promote tissue repair. Macrophage-derived EVs have been shown to enhance PI3K/Akt signaling and promote primordial follicle recruitment in aged ovaries (42). The key oocyte endpoint for EV-based therapies is improved meiotic competence and developmental potential. Current evidence is preclinical, with follicle activation as the primary reported endpoint and direct oocyte assessment still limited; human translational data are lacking.

EV-based therapies offer targeted delivery and reduced immunogenicity compared with cell-based therapies. As the cargo of EVs becomes better characterized, it may be possible to develop EV-based interventions that specifically improve oocyte quality through immunomodulatory mechanisms. **Table 3** summarizes the major molecular pathways involved in ovarian immune aging and the therapeutic strategies targeting these pathways for oocyte quality preservation.

**Table 3:** Molecular pathways and therapeutic targets in ovarian immune aging and oocyte quality decline.

Mechanisms	Key mediators	Effects on oocyte quality	Therapeutic strategies	References
Inflammaging	Chronic low-grade inflammation; elevated IL-6, TNF-α; senescent cell accumulation (SASP)	Direct endpoints: (1) Spindle microtubule destabilization and chromosome misalignment; (2) Increased aneuploidy rates; (3) Impaired polar body extrusion; (4) Reduced fertilization capacity; (5) Oxidative DNA damage in oocytes; (6) Premature oocyte apoptosis via caspase-3/9 activation. Indirect endpoints: Ovarian fibrosis impairing follicle development.	Senotherapeutic strategies to reduce SASP and fibrosis. Immune modulation to limit chronic inflammation.	(62, 64, 77, 79, 85–87)
NLRP3 Inflammasome	Activated by mitochondrial ROS and oxidative stress; promotes IL-1β and IL-18 production.	Direct endpoints: (1) Meiotic spindle disruption and metaphase II arrest; (2) Mitochondrial membrane potential (ΔΨm) collapse; (3) Increased oocyte ROS levels; (4) Impaired cytoplasmic maturation (cortical granule distribution defects); (5) Reduced oocyte survival and increased fragmentation; (6) Decreased blastocyst formation rates. Indirect endpoints: Inflammatory microenvironment.	Pharmacological NLRP3 inhibitors prevent ovarian aging in mouse models (preclinical only; human efficacy data lacking). Inhibition reduces inflammatory burden without global immunosuppression in animal studies.	(76, 80, 82–84)
Macrophage Polarization	M1 (proinflammatory) vs. M2 (anti-inflammatory/reparative) phenotypes.	Direct endpoints: (1) Elevated TNF-α from M1 macrophages disrupts metaphase II spindle organization; (2) IL-6 exposure associated with spindle abnormalities; (3) M2-derived EVs enhance oocyte mitochondrial function and meiotic competence; (4) Altered oocyte metabolomic profiles; (5) Impaired cumulus-oocyte complex expansion. Indirect endpoints: Impaired angiogenesis compromising oocyte nutrient supply.	Promote M2 polarization via IL-10, small molecules, or MSC/EV therapies. Macrophage-derived EVs enhance follicle activation in aged ovaries.	(41, 42, 44, 45, 51, 53, 61)
Treg/Th17 Balance	Treg (tolerance) vs. Th17 (proinflammatory) differentiation; regulated by local cytokines and metabolism	Direct endpoints: (1) Th17-derived IL-17 impairs oocyte maturation rates; (2) Treg-derived IL-10 promotes oocyte developmental competence in co-culture models; (3) Altered Treg/Th17 balance correlates with reduced oocyte yield and fertilization outcomes. Indirect endpoints: Granulosa cell apoptosis and follicle loss.	Low-dose IL-2 to expand Treg populations. Metabolic modulators to influence T cell differentiation (e.g., Rheb/mTOR inhibition).	(19, 55, 67, 69, 101)
Mitochondrial Dysfunction	Damaged mitochondria produce excess ROS; activate NLRP3; impair energy production in oocytes and immune cells.	In preclinical models, direct endpoints: (1) Decreased ATP production compromising meiotic spindle assembly; (2) Mitochondrial DNA (mtDNA) copy number reduction; (3) mtDNA mutations and deletions affecting oocyte competence; (4) Impaired calcium homeostasis and cortical granule exocytosis; (5) Compromised embryonic development post-fertilization; (6) Accelerated oocyte aging and reduced developmental potential. Indirect endpoints: Triggers inflammatory responses via NLRP3.	Mitochondria-targeted antioxidants or metabolic modulators under investigation. NLRP3 inhibition indirectly addresses mitochondrial-driven inflammation.	(80, 82, 83, 85)
Ferroptosis	Iron-dependent lipid peroxidation; implicated in loss of regulatory immune populations.	Direct endpoints: (1) Lipid peroxidation-induced oocyte membrane damage; (2) Reduced oocyte maturation and fertilization rates; (3) Increased oocyte degeneration and fragmentation; (4) Depletion of oocyte glutathione (GSH) pools; (5) Iron accumulation and oxidative membrane damage. Indirect endpoints: May exacerbate immune dysregulation via loss of regulatory populations.	Ferroptosis inhibitors (e.g., ferrostatin-1) show promise in preclinical models for oocyte preservation.	(4, 90–92)

Effects on oocyte quality are graded by evidence level: “direct” denotes oocyte-intrinsic endpoints measured in the cited studies; “indirect” denotes follicular or stromal endpoints from which oocyte effects are inferred. Unless otherwise stated, direct oocyte endpoints are derived from preclinical models, and human evidence is correlative.

## Conclusions and future directions

The evidence reviewed here indicates that the ovarian immune microenvironment is associated with oocyte quality, although the strength of this relationship varies across mechanisms and is, for many pathways, supported primarily by preclinical or correlative evidence. From prenatal colonization of the developing ovary by macrophages to age-related immune dysregulation that has been associated with impaired oocyte competence, immune signals participate in the processes that regulate oocyte development, with the caveat that direct evidence in human oocytes remains limited for most mechanisms.

Ovarian immune homeostasis appears to be initiated during prenatal colonization (13, 26), where early-life perturbations can program long-term reproductive dysfunction (28, 30). Within this dynamic milieu, macrophages serve as important modulators of ovarian function, with pro-inflammatory macrophage states (M1-like) governing follicle activation while M2-like populations sustain tissue repair and vascular integrity. T lymphocytes orchestrate immune tolerance, and alterations in Treg subsets—including Treg/Th1 shifts in primary POI and Treg/Th17 imbalances reported in other infertility contexts—have been associated with both physiological reproductive aging and pathological POI in correlative human studies; causal relationships and tissue-specific mechanisms require further investigation. Importantly, POI is a heterogeneous clinical syndrome encompassing genetic, iatrogenic, autoimmune, and idiopathic subtypes, and immune mechanisms are relevant primarily to a subset of cases; overlap with physiological ovarian aging should therefore not be interpreted as evidence that the two conditions share a common primary mechanism. As the ovarian microenvironment ages, a self-reinforcing cycle driven by inflammaging and the SASP may contribute to a proinflammatory niche that has been associated, primarily in preclinical models, with compromised oocyte meiotic competence and developmental potential. At the molecular level, the activation of the NLRP3 inflammasome, potentiated by mitochondrial dysfunction and oxidative stress, has been implicated in preclinical models as a potential mechanistic link between immune dysregulation and declining oocyte quality; human evidence remains correlative.

More recently, oocyte-intrinsic cGAS-STING activation and cGAMP transfer to granulosa cells have been demonstrated in preclinical models as a defined innate immune sensing axis, whereas the broader ferroptosis–DAMP–TLR4–NF-κB feedback loop remains a proposed integrative model rather than an experimentally established ovarian circuit.

These findings suggest that targeting the ovarian immune microenvironment—through strategies such as modulating macrophage polarization, restoring the Treg/Th17 axis, inhibiting NLRP3 activation, or employing MSC and EV therapies—is a candidate approach for fertility preservation that requires further investigation. Most of these strategies remain at the preclinical stage, with oocyte competence rarely assessed as a primary endpoint, and require clinical validation before translation into routine practice.

Despite the advances synthesized here, significant gaps in knowledge remain. First, the functional heterogeneity of ovarian immune populations has not been fully characterized; emerging single-cell and spatial transcriptomic approaches are beginning to address this. Second, the spatiotemporal dynamics governing immune cell recruitment and activation throughout follicular progression remain elusive. Third, the mechanistic underpinnings of direct immune-oocyte communication—particularly the relative contributions of cytokine signaling versus EV-mediated transfer—require rigorous elucidation. Fourth, the evidence base linking ovarian immune signals to post-fertilization events remains sparse, representing a priority area for future investigation. Finally, the translational potential of immunomodulatory strategies for fertility preservation has yet to be clinically validated; well-designed clinical trials are urgently needed.

### From oocyte-centric to microenvironment-aware models

The evidence reviewed here supports a conceptual shift from oocyte-centric to microenvironment-aware models, recognizing the ovarian immune niche as a potential contributor to oocyte competence. This perspective offers new directions for research, encouraging investigation of immune-oocyte interactions across the reproductive lifespan, which complements broader surveys (21, 22) by organizing evidence according to functionally relevant endpoints centered on oocyte quality.

Compared with recent reviews on ovarian immune microenvironment, the present review is organized around oocyte-specific endpoints. Rather than cataloging ovarian immune cells, we distinguish immune mechanisms with direct, measurable effects on the oocyte from those acting indirectly through follicular or stromal endpoints, and indicate which remain supported only by preclinical or correlative evidence. This endpoint-driven organization is intended to complement, not duplicate, broader surveys of ovarian immunology.

### The next frontier in reproductive immunology

The integration of immunology and reproductive biology remains in its nascent stages; however, recent progress has illuminated several promising avenues that may collectively advance the field. Looking forward, priority research directions include: (i) mechanistic validation of candidate immune targets in human ovarian tissue and oocytes, a prerequisite for any therapeutic development; (ii) longitudinal immune profiling to determine whether immune signatures can eventually be developed as biomarkers of oocyte competence or ovarian aging; (iii) characterization of how prenatal and early-life immune exposures shape lifelong ovarian function, as a basis for understanding rather than for prophylaxis; and (iv) controlled clinical testing of immunomodulatory strategies in defined populations, such as women undergoing gonadotoxic cancer therapy, with oocyte competence as a prespecified endpoint. In conclusion, the immune system is an integral component of mammalian reproduction. A deeper mechanistic understanding of immune regulation of oocyte development and quality may inform future research directions (21, 22, 63). While the immune mechanisms discussed herein—including macrophage polarization, NLRP3 inflammasome activation, and SASP—are observed across multiple aging tissues, their ovarian manifestation is shaped by the unique reproductive context of cyclical hormonal fluctuations, a finite and non-renewable germ cell pool, and compartmentalized immune surveillance. Systematic cross-tissue comparisons represent an important avenue for future investigation.

Despite recent progress, several fundamental questions remain unresolved. First, most mechanistic claims rest on preclinical models, and direct validation in human ovarian tissue and oocytes is needed. Second, immune profiling in the ovary has been largely cross-sectional; longitudinal studies are required to determine whether immune changes precede, accompany, or follow oocyte decline. Third, ovarian reserve and oocyte quality are related but distinct endpoints, and studies should specify which is being assessed. Fourth, the spatial and temporal states of ovarian immune cells remain poorly defined, and single-cell and spatial approaches should be extended to human tissue. Fifth, candidate immunomodulatory interventions require controlled clinical testing with oocyte competence as a prespecified endpoint before any translational claim can be made. Among these, the origin and maintenance of the ovarian resident macrophage pool—whether retained and self-maintained or replaced by circulating monocytes after transplantation—remains one specific and unresolved question (26, 31). Addressing these questions will determine whether the ovarian immune microenvironment can be safely and effectively targeted to preserve oocyte quality in humans. 

## Glossary

ASC: Apoptosis-associated Speck-like Protein Containing a CARD
CD11c: Cluster of Differentiation 11c
CTLA-4: Cytotoxic T-Lymphocyte-Associated Protein 4
DAMP: Damage-Associated Molecular Pattern
DC: Dendritic Cell
ECM: Extracellular Matrix
EGF: Epidermal Growth Factor
EV: Extracellular Vesicle
FOXO3: Forkhead Box O3
HGF: Hepatocyte Growth Factor
ICI: Immune Checkpoint Inhibitor
IFN-γ: Interferon-Gamma
IGF: Insulin-like Growth Factor
IL-1β: Interleukin-1 Beta
IL-6: Interleukin-6
IL-10: Interleukin-10
IL-18: Interleukin-18
IVF: *In Vitro* Fertilization
JAK: Janus Kinase
M1: Classically Activated Macrophage
M2: Alternatively Activated Macrophage
MSC: Mesenchymal Stem Cell
mTOR: Mammalian Target of Rapamycin
NK: Natural Killer Cell
NLRP3: NOD-like Receptor Family Pyrin Domain Containing 3 (historical name NACHT, LRR, and PYD domains-containing protein 3)
PD-1: Programmed Cell Death Protein 1
PD-L1: Programmed Death-Ligand 1
PI3K: Phosphoinositide 3-Kinase
POI: Premature Ovarian Insufficiency
PRP: Platelet-Rich Plasma
ROS: Reactive Oxygen Species
SASP: Senescence-Associated Secretory Phenotype
STAT3: Signal Transducer and Activator of Transcription 3
TGF-β: Transforming Growth Factor-Beta
Th1: T Helper 1 Cell
Th17: T Helper 17 Cell
TNF-α: Tumor Necrosis Factor-Alpha
Treg: Regulatory T Cell
VEGF: Vascular Endothelial Growth Factor
